# In Vitro and In Vivo Biocompatible and Controlled Resveratrol Release Performances of HEMA/Alginate and HEMA/Gelatin IPN Hydrogel Scaffolds

**DOI:** 10.3390/polym14204459

**Published:** 2022-10-21

**Authors:** Jovana S. Vuković, Vuk V. Filipović, Marija M. Babić Radić, Marija Vukomanović, Dusan Milivojevic, Tatjana Ilic-Tomic, Jasmina Nikodinovic-Runic, Simonida Lj. Tomić

**Affiliations:** 1 University of Belgrade, Faculty of Technology and Metallurgy, Karnegijeva 4, 11000 Belgrade, Serbia; 2 University of Belgrade, Institute of Molecular Genetics and Genetic Engineering, Vojvode Stepe 444a, 11000 Belgrade, Serbia; 3 Advanced Materials Department, Jožef Stefan Institute, Jamova Cesta 39, 1000 Ljubljana, Slovenia

**Keywords:** scaffolding polymeric biomaterials, interpenetrating hydrogel networks 2-hydroxyethyl methacrylate/alginate and 2-hydroxyethyl methacrylate/gelatin, in vitro and in vivo biocompatibility, controlled resveratrol release

## Abstract

Scaffold hydrogel biomaterials designed to have advantageous biofunctional properties, which can be applied for controlled bioactive agent release, represent an important concept in biomedical tissue engineering. Our goal was to create scaffolding materials that mimic living tissue for biomedical utilization. In this study, two novel series of interpenetrating hydrogel networks (IPNs) based on 2-hydroxyethyl methacrylate/gelatin and 2-hydroxyethyl methacrylate/alginate were crosslinked using N-ethyl-N′-(3-dimethyl aminopropyl)carbodiimide hydrochloride (EDC) and N-hydroxysuccinimide (NHS). Characterization included examining the effects of crosslinker type and concentration on structure, morphological and mechanical properties, in vitro swelling, hydrophilicity as well as on the in vitro cell viability (fibroblast cells) and in vivo (*Caenorhabditis elegans*) interactions of novel biomaterials. The engineered IPN hydrogel scaffolds show an interconnected pore morphology and porosity range of 62.36 to 85.20%, favorable in vitro swelling capacity, full hydrophilicity, and Young’s modulus values in the range of 1.40 to 7.50 MPa. In vitro assay on healthy human fibroblast (MRC5 cells) by MTT test and in vivo (*Caenorhabditis elegans*) survival assays show the advantageous biocompatible properties of novel IPN hydrogel scaffolds. Furthermore, in vitro controlled release study of the therapeutic agent resveratrol showed that these novel scaffolding systems are suitable controlled release platforms. The results revealed that the use of EDC and the combination of EDC/NHS crosslinkers can be applied to prepare and tune the properties of the IPN 2-hydroxyethyl methacrylate/alginate and 2-hydroxyethyl methacrylate/gelatin hydrogel scaffolds series, which have shown great potential for biomedical engineering applications.

## 1. Introduction

Scaffolding polymeric biomaterials are vital materials in the field of biomedical engineering. Their functions mimic native tissue biofunctionalities by providing structural stability and favorable environmental conditions for tissue regeneration [1,2]. Three-dimensional scaffolds have been assessed for a wide variety of applications ranging from bone [3], nerve [4], muscle, and tendon/ligament [5] tissue regeneration. Various natural and synthetic polymers and their combinations with different types of inorganic material (hybrids) scaffolds have been studied and recognized as advantageous scaffolding biomaterials [6]. Among natural materials, gelatin and alginate are attractive polymers due to their natural origin, favorable properties, and availability [7,8]. The composition of scaffolding materials can be finely tuned by varying the ratio of synthetic and natural components [9,10] in order to obtain properties which are as similar to natural tissue as possible. The scaffolds used in biomedical tissue engineering must possess the required properties, including prominent biocompatibility, stability and mechanical strength able to withstand the different loadings and stresses, and an interconnected porous structure with appropriate pore size, in order to produce a good outcome for tissue interaction assays [11,12]. The permeability of the three-dimensional constructs used in engineered tissue for nutrient flow and waste disposal is an important factor in the size and interrelationship of available spaces [13,14].

Hydrogels are 3D hydrophilic cross-linked polymeric networks with the ability to maintain appropriate content of fluid/water with predictable biocompatibility, biodegradability, suitable stimuli responsiveness, and mechanical performances [15]. Their properties can be adjusted to meet the diverse applicable demands of the variation of hydrophilic and hydrophobic proportion or the addition of an active recognition motif [16,17]. Hydrogels are considered to be the most promising prospective alternative biomaterials for tissue due to their exceptional mechanical properties [18], features which make them superb materials for controlled drug release or external stimuli recognition in biomedical applications [17].

Interpenetrating polymeric networks (IPNs) are a specific class of hydrogels and a unique combination of cross-linked polymers in which at least one network is synthesized and/or cross-linked in the presence of the other [19,20,21]. IPNs are also known as entanglements of polymeric networks that are specifically held together only by permanent topological interactions. IPN formation can improve the performance of hydrogels by combining favorable individual properties of two or more polymers. In some cases, IPNs show completely new features that are not perceived in either individual network [19,20,21]. The development of interpenetrating network polymers is attractive because their three-dimensional structure ensures free space for easy drug loading [19,20,21]. Various properties of IPNs, such as porosity, bioadhesiveness, elasticity, swelling and stimuli-sensitive properties, can be tuned by the appropriate choice of network forming polymers and suitable cross-linking agents and their formulations [19,20,21]. IPN hydrogels possess specific, tunable properties and have a significant role as scaffolding biomaterials in biomedical engineering [22,23,24,25]. The development of interpenetrating polymer network hydrogels as scaffolding biomaterials can provide cells with an adhesive extracellular matrix-like 3D microenvironment possessing the mechanical integrity to withstand physiological forces. These hydrogels can be synthesized from biologically derived or synthetic polymers, the former polymer offering preservation of adhesion, degradability, and microstructure, and the latter offering tunability and superior mechanical properties [26].

Alginate is a natural biocompatible polysaccharide widely used in biomedical and pharmaceutical fields [27,28,29,30,31,32,33]. Alginates are anionic polysaccharides derived from brown algae cell walls (*Macrocystis pyrifera*, *Laminaria hyperborea*, *Ascophyllum nodosum*) [34], whose properties have been investigated since their discovery more than a hundred years ago [27,28,29]. They were shown to be biocompatible both in vitro and in vivo and biodegradable in the human body [30,31,32,33], and they can be utilized to create IPN hydrogel scaffolds for biomedical uses [21,35,36,37]. Gelatin is a biodegradable protein polymer and is widely used in the biomedical field [38,39,40,41,42,43,44]. It is obtained through the denaturation or partial hydrolysis of collagen and is a major biomacromolecule component of the natural extracellular matrix (ECM) of several tissues such as the cornea, epithelium, skin, ligaments, tendon, heart, cartilage, bone, and blood vessels [38,39,40,41,42,43,44]. Gelatin bears protease cleavage sites and cell-interactive functional groups, the Arg–Gly–Asp (RGD) sequence located in the adhesion proteins of the natural ECM [38,39,40,41,42,43,44], which enhances cell adhesion. Scaffolds based on gelatin can be easily degraded by proteases [44], which is essential to make space for the formation of new ECM by cells. When used as a component in hydrogel scaffold composition, gelatin improves suitability for biomedical engineering applications [38,39,40,41,42,43,44] and is favorable for the preparation of IPN hydrogel scaffolds [37,45]. 2-Hydroxyethyl methacrylate (HEMA) is an outstanding monomer, and HEMA-based polymeric biomaterials have been successfully applied in ophthalmology, wound dressings, hemodialysis membranes, controlled drug release systems, and tools for tissue reconstructive surgery [46,47,48,49,50,51,52]. These applications were enabled by advantageous properties, such as hydrophilicity, absorption potential, biocompatibility, inertness, and tissue-like mechanical features [53,54,55].

A significant number of natural polymers have been investigated for specific drug delivery applications, such as targeted, cancer, topical drug delivery, and wound healing [56]. The benefits of their natural origin enhance the healing process of the specific site due to unique bioactivities and interactions with the biologically relevant receptors. Transdermal drug delivery systems with polymeric components of natural origin have been widely investigated over the past decade, including gelatin, alginate, hyaluronic acid, pectin, and pullulan [57].

The nutraceutical agent resveratrol (RSV) belongs to the stilbene family of phytoalexins and is produced by several plants either in response to injury or when the plant is under attack by pathogens (bacteria or fungi). RSV has physiological functions and possesses a great beneficial effects, such as being cardioprotective, antioxidant, anti-inflammatory, anti-hyperlipidemic, anti-obesity, immunomodulatory, and anti-cancer properties [58,59,60,61,62,63,64,65,66,67,68,69,70,71]. Resveratrol has been shown to suppress fibroblast proliferation and induce apoptosis, which inhibits fibrogenesis in keloids [72]. Resveratrol further reduced the production of pro-inflammatory factors and elevated the level of SIRT1 in a severe burn [73]. Therefore, it can be useful as a bioactive agent in controlled release systems.

Our study explores the design of novel biocompatible, bioactive scaffolding hydrogels for biomedical applications. Interpenetrating hydrogel networks were prepared via a simple route using synthetic monomeric component 2-hydroxyethyl methacrylate and polymers of natural origin (gelatin and alginate) crosslinked by N-(3-dimethyl aminopropyl)-N′ ethyl carbodiimide hydrochloride and N-hydroxysuccinimide. Hydrogel scaffolds’ functional performances important for biomedical engineering applications were tested. The extensive biological testing was conducted through both in vitro and in vivo assays using a human fibroblast cell line and microworm *Caenorhabditis elegans*. In order to investigate the synthesized hydrogels as novel transdermal drug delivery systems, the in vitro controlled release process of resveratrol was monitored over a short period of time and release parameters were obtained for these resveratrol/polymeric scaffold platforms according to four release models.

## 2. Materials and Methods

### 2.1. Materials

2-Hydroxyethyl methacrylate (H, 99%) monomer and polymers of natural origin—gelatin from porcine skin (G, gel strength 300, Type A) and alginate (A)—were supplied from Sigma-Aldrich, Burghausen, Germany. Cross-linking agents N-hydroxysuccinimide (NHS) and N-ethyl-N′-(3-dimethyl aminopropyl)carbodiimide hydrochloride (EDC) and polymerization agensts potassium persulfate (PPS) and N,N,N′,N′-tetramethylene diamine (TEMED) were purchased from Sigma-Aldrich, St. Louis, MO, USA. RPMI-1640 medium and supplements for cell proliferation as well as 3-(4,5-dimethylthiazol-2-yl)-2,5-diphenyltetrazolium bromide (MTT) reduction assay components were purchased from Sigma-Aldrich, St. Louis, MO, USA. All polymerizations and buffer preparations were performed in deionized water. Resveratrol (RSV) as a bioactive agent was purchased from Sigma-Aldrich, Germany.

### 2.2. Hydrogel Syntheses

Interpenetrating hydrogel networks consisting of HEMA (H), alginate (A), and gelatin (G) were synthesized using free-radical polymerization/crosslinking at −18 °C for 24 h. H/A and H/G = 0.8/0.2 were dissolved in deionized water and stirred at room temperature. The next step was the addition of agents for free-radical polymerization/crosslinking (Table 1). The reaction mixture was transferred to a Petri dish and placed to perform the reaction. Samples were soaked in deionized water for 7 days. Water was changed daily. Swollen gels were frozen and freeze-dried. Sample marks are listed in Table 1.

### 2.3. Hydrogel Scaffold Characterization

#### 2.3.1. Fourier Transform Infrared Spectroscopy (FTIR)

Hydrogel composition was analyzed using FTIR spectra, recorded on a Thermo-Scientific Nicolet 6700 FTIR diamond crystal spectrometer, using the attenuated total reflectance (ATR) sampling technique. FTIR spectra were recorded over the wavelength range of 700–4000 cm^−1^.

#### 2.3.2. Scanning Electron Microscopy (SEM)

Morphological analysis of the scaffolds was performed with SEM (Jeol JSM-7600 F). Samples, which were previously freeze-dried (using Martin Christ–Alpha 1–2 LDplus), were cut into slices, fixed on a holder using carbon tape, and sputtered with gold (using BAL-TEC SCD 005), and lyophilized in a vacuum chamber (VC 50 SalvisLab Vacucenter).

#### 2.3.3. Mechanical Properties Testing

The mechanical properties of hydrogel scaffolds were analyzed using Galdabini Quasar 50, Italy, by applying a uniaxial compression with a 100 N load cell at room temperature. The Young’s modulus (*E*) of the hydrogels was calculated from the linear part of the stress/strain curve. Each measurement was repeated three times, and the final value of Young’s modulus was given as the average value.

#### 2.3.4. Water Contact Angle Measurements

The static water contact angle measurement was realized by the sessile drop method, putting approximately 1 μL drop of MilliQ water on the hydrogel’s surface. The measurements were performed using a Theta Lite–Biolin Scientific Contact angle meter in a measuring range from 0 to 180 deg. and accuracy +/− 0.1 deg., +/− 0.01 mN/m with a camera of 640 × 480 resolution and a maximum measuring speed of 60 fps.

#### 2.3.5. Porosity Measurements

The porosity of hydrogels was determined by the solvent replacement method [74]. Glycerol (ρ = 1.2038 g/cm^3^) was used as a wetting medium. Dried hydrogels were submerged in glycerol for 24 h and weighed after removing excess glycerol from the surface:

$$\mathrm{Porosity}=\frac{\left(m_{\mathrm{glycerol}}-m_{i}\right)}{\rho V}\times 100$$

where m_i_ is the initial weight of the dry hydrogel, m_glycerol_ is the weight of the hydrogel with glycerol, ρ is the density of glycerol, and V is the volume of the hydrogel sample.

#### 2.3.6. In Vitro Swelling Study

An in vitro swelling study was performed in order to obtain swelling profiles for all of the hydrogel samples using the gravimetric method. Dried hydrogel discs were immersed in phosphate buffer (pH of 7.40 at 37 °C). Discs were taken out of the buffer at selected times and dried by removing excess water and weight. The degree of swelling was determined by the equation:

$$q=\frac{m_{s}-m_{i}}{m_{i}}$$

where *m_i_* is the initial weight of the dry gel and *m_s_* is the weight of the swollen sample, measured at selected time intervals, at the time of measuring [75,76].

### 2.4. Biocompatibily Probes

#### 2.4.1. In Vitro Cytotoxicity Assay

The cytotoxic activities of the samples were measured using the methods described previously [77]. Cytotoxicity/antiproliferative activity was measured using MTT assay by the following procedure: 100 mg of the sample hydrogel was aseptically ground and incubated in 10 mL of RPMI-1640 medium for 72 h at 37 °C. The samples were shaken at 180 rpm. Suspensions were briefly centrifuged for 10 min at 5000 rpm (Eppendorf Centrifuge 5804R) and the supernatants were used in different concentrations. Human lung fibroblast (MRC5 cells) was maintained as monolayer cultures in RPMI-1640 medium enriched with 10% (v/v) heat-inactivated fetal bovine serum (FBS), 100 U/mL penicillin, and 100 µg/mL streptomycin at 37 °C in a humidified atmosphere in the presence of 5% CO_2_. The cells were plated in a 96-well flat-bottom plate at a concentration of 1 × 10^4^ cells per well, and after 24 h of cells incubation, media containing increasing concentrations of material extracts—12.5%, 25%, 50% and 100% (v/v)—were added to the cells. Control cultures contained 200 μL of growth medium. After 48 h of incubation, cell cytotoxicity was determined using 3-(4,5-dimethylthiazol-2-yl)-2,5-diphenyl tetrazolium bromide (MTT) reduction assay. Cell proliferation was calculated by measuring the absorbance at 540 nm on a multiplate reader (Epoch 2000, BioTek, Winooski, VT, USA). The MTT assay was performed twice in quadruplicate, and the results were presented as a percentage of the control (untreated cells) that was arbitrarily set to 100%. The cell viability rate (%) was calculated as (OD of the treated group/OD control group) × 100.

#### 2.4.2. *Caenorhabditis elegans* Survival Assay

*Caenorhabditis elegans* N2 (glp-4; sek-1) (*C. elegans*) was propagated under standard conditions, synchronized by hypochlorite bleaching, and cultured on nematode growth medium using *E. coli* OP50 as a food source, as described previously [78]. The *C. elegans* survival assay was carried out as described previously with minor modifications [79]. The procedure is that synchronized worms (L4 stage) were suspended in a medium containing 95% M9 buffer (3 g of KH_2_PO_4_, 6 g of Na_2_HPO_4_, 5 g of NaCl, and 1 mL of 1 M MgSO_4_ x 7H_2_O in 1 L of water), 5% LB broth (Oxoid), and 10 μg of cholesterol (Sigma-Aldrich) per mL. Material extracts were prepared as described previously in Section 2.4.1. Fifty μL of this suspension of nematodes (25–35 nematodes) were transferred to the wells of a 96-well microtiter plate where 50 μL of sterile medium were added to the control while 50 μL media containing increasing concentrations of material extracts—12.5%, 25%, 50% and 100% (v/v)—was added to the tested wells. The plates were incubated at 25 °C for 48 h. The fraction of dead worms was determined by counting the number of dead worms and the total number of worms in each well using a stereomicroscope (SMZ143-N2GG, Motic, Germany). The material extracts were tested at least three times in each assay, and each assay was repeated at least two times (n  ≥  6).

### 2.5. In Vitro Controlled Resveratrol Release Study

An in vitro controlled release study was performed in a buffer of pH 7.40 at 37 °C. Resveratrol was loaded into the hydrogels using the swelling–diffusion method. The release process was performed in a basket stirrer containing 15 mL of release medium. The amount of the released resveratrol was measured by taking the absorbance of the solution containing released resveratrol at regular time intervals using a UV/Vis spectrophotometer (Shimadzu UV/Vis Spectrophotometer UV-1800, Kyoto, Japan) at a λ_max_ value of 305 nm.

## 3. Results and Discussion

The novel two series of 2-hydroxyethyl methacrylate/alginate and 2-hydroxyethyl methacrylate/gelatin interpenetrating hydrogel networks (IPN) were designed as multifunctional, bioactive scaffolding materials for multiple biomedical purposes, especially for controlled release of the bioactive agent resveratrol. The first series of IPNs were made using monomeric 2-hydroxyethyl methacrylate and alginate by varying the concentrations and types of crosslinkers (EDC and EDC/NHS) using free-radical polymerization/crosslinking reactions. The second series of IPN was created using monomeric 2-hydroxyethyl methacrylate and gelatin by varying the concentration and type of crosslinkers (EDC and EDC/NHS) using free-radical polymerization/crosslinking reactions.

### 3.1. Structural Characteristics of Hydrogel Scaffolds

**Figure 1:**
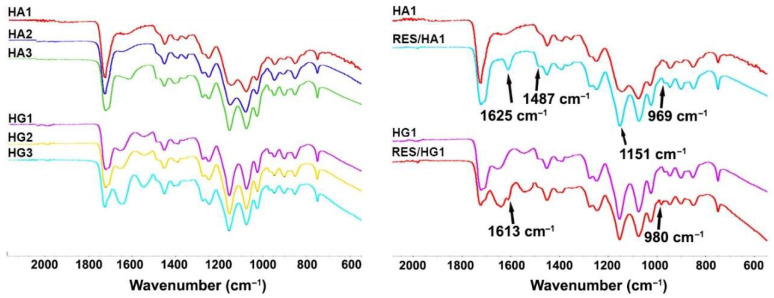
FTIR spectra of IPN HA and HG hydrogel scaffolds series.

Spectral analysis (FTIR spectra) gave an insight into the structural characteristics of the obtained hydrogel scaffolds. The FTIR spectra of two series of IPN hydrogels based on 2-hydroxyethyl methacrylate/alginate and 2-hydroxyethyl methacrylate/gelatin (HA1, HA2, HA3, HG1, HG2, HG3) and sample HA1 and HG1 loaded with the bioactive agent resveratrol are shown in Figure 1. The HEMA component shows signals for O–H stretching vibration at approximately 3431 cm^−1^, strong C=O vibration at 1716 cm^−1^, and C–H stretching vibrations at 2929 cm^−1^ and 2885 cm^−1^ [80,81]. The FTIR spectra of the obtained HG1, HG2, HG3, HA1, HA2, and HA3 samples exhibit above listed HEMA signals, gelatin (N–H stretching vibration around 3354 cm^−1^, C–H stretching at 3003 cm^−1^, C=O stretching at 1701 cm^−1^ for amide I, N–H definition at 1630 cm^−1^ for the amide II) and alginate (1278 cm^−1^ C–O stretching, 1160 cm^−1^ C–C stretching, 1017 cm^−1^ C–O–C stretching) vibrations and the type and ratio of the component influenced on the intensity of the peaks [81,82]. The FTIR spectra of samples HA1 and HG1 loaded with resveratrol (Figure 1) reveal additional absorption bands at around 1620 cm^−1^ (aromatic C–C double-bond stretching), 1487 cm^−1^ (olefinic C–C olefinic stretching), 1151, and 980 cm^−1^ (typical trans olefinic band) [83].

### 3.2. Morphology of Hydrogel Scaffold

**Figure 2:**
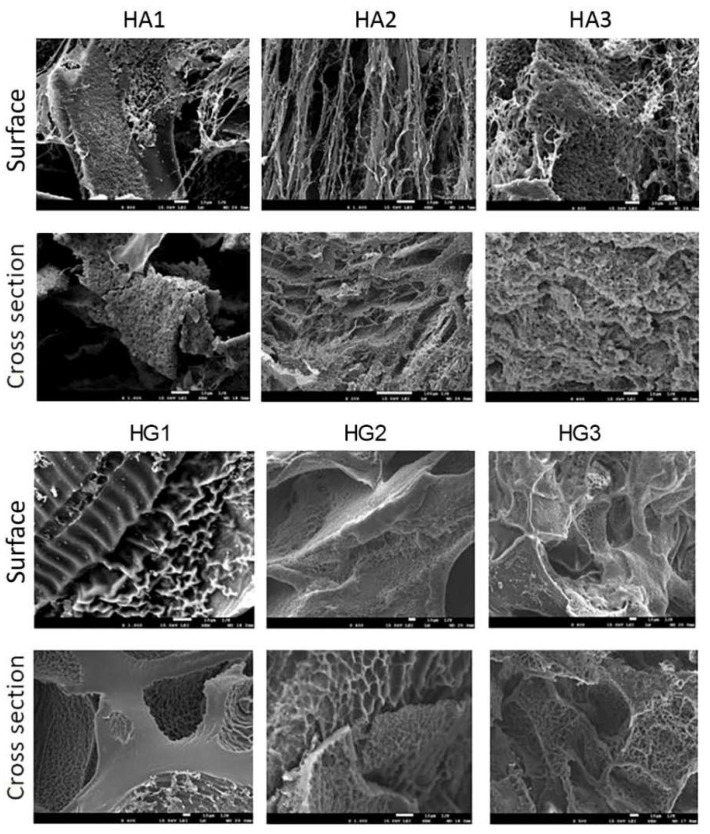
SEM micrographs of obtained HA and HG IPN hydrogel scaffold series (bar represents 10 μm).

An advantageous scaffolding biomaterial should provide a suitable 3D porous structure with interconnected pores to promote cell adhesion, regeneration, and growth, as well as oxygen, nutrient species, and waste flow [84]. The porous structure is also crucial for the efficient loading and release of the active agent. Therefore, the morphological structure of the scaffolding biomaterials should be specifically engineered and tuned. The cross-sectional and surface morphology of the synthesized HA and HG IPN hydrogel scaffold series was observed by SEM. The micrographs are presented in Figure 2.

The micrographs of all samples indicated the presence of favorable interconnected porous morphology. Layered morphology was observed, which affects the porosity, mechanical properties, and swelling properties. The main pattern is an ellipsoidal to spherical pore structure in samples where an EDC crosslinker was used (HA1, HA2, HG1, and HG2). When EDC/NHS were used as crosslinkers, the morphology of the samples has the appearance of a honeycomb template (HA3 and HG3). It is important that the interconnected pore structure of hydrogel scaffolds was created, which indicates the potential of biomedical applications. The presence of micropores and interconnectivity of pores in the hydrogel morphology supports cell growth, nutrient flow, and metabolic waste excretion within the polymeric networks.

### 3.3. Porosity of Hydrogel Scaffold

The suitable porosity of the scaffolding biomaterials is a significant parameter for the successful support of cell adhesion, regeneration, and growth. The important criterion is to target the porosity of the natural tissue [85]. Due to the porosity having a substantial effect on the mechanical properties of the scaffolding biomaterials, it is important to make a balance between the mechanical properties and porosity of the biomaterial to suit its final application. The obtained results of the porosity measurements are presented in Table 2 indicating that the concentration and type of crosslinker affect porosity. Porosity values are from 62.36% to 85.20%, favorable for biomedical applications (Table 2). For the HA series, it can be seen that the porosity decreases when the concentration of EDC is doubled, which is expected. When the combination of EDC/NHS crosslinkers is used, the highest porosity is obtained. In the case of the HG series, the lowest porosity was obtained when a lower concentration of EDC was used. When the combination of EDC/NHS crosslinkers is used, a mean value of porosity is obtained. It can be concluded that EDC and EDC/NHS have an impact on crosslinking these series of hydrogels in different ways.

### 3.4. Mechanical Properties of Hydrogel Scaffolds

The advantageous scaffold should be able to fully mimic the strength, stiffness, and mechanical performances of natural tissue to withstand physiological loads. Scaffolding biomaterial should match the mechanical features of the targeted natural tissue to realize its function as a biomechanical construct [86]. The elastic modulus of the scaffolding biomaterials is a very important parameter for evaluating their biomedical potential application. Thus, the biomechanical properties of the HG and HA hydrogel scaffolds series were analyzed by Young’s modulus values (Table 2). The values of Young’s modulus (E) of the HG and HA series hydrogel scaffolds depend upon the crosslinker type and concentration. For HA series scaffolds, when a higher EDC concentration was used, the modulus values decreased from 7.50 to 3.50 MPa. When combined EDC/NHS crosslinkers were used, the module value is 5.00 MPa (in the middle of the series values range). When considering the influence of EDC concentrations on the HG series, it is noticed that with the increase in EDC concentration, the modulus increases from 1.40 to 2.50 MPa. The largest modulus is obtained when using a combination of EDC/NHS crosslinkers (3.50 MPa). The use of these combinations of concentration and crosslinker type has been shown to have different effects on the HA and HG series hydrogel scaffolds.

### 3.5. Swelling Properties of Hydrogel Scaffolds

**Figure 3:**
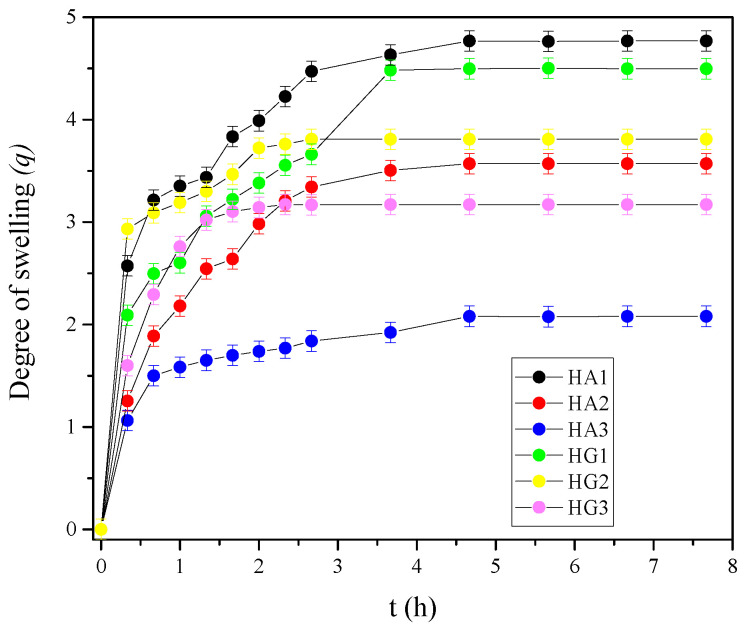
In vitro swelling profiles for HA and HG hydrogel scaffolds series.

The swelling ability is an influential indicator for the potential applications of a hydrogel in biomedical tissue engineering, and drug release systems [87]. The swelling performances of hydrogel scaffolds indicate the absorption capacities of scaffolds, which is a very important property for tissue regeneration. In vitro swelling studies were performed in a buffer of pH 7.40 at 37 °C to mimic the physiological milieu. Data for scaffold swelling are presented in Figure 3 as the degree of swelling (q) value versus time. The initial fast-swelling behavior [88], typical for highly hydrophilic and porous hydrogel biomaterials, can be observed for all samples from a very steep slope of the swelling curves at the beginning of the swelling process. Swelling depends on composition (influence of alginate and gelatin), the type of crosslinkers and their concentration. The IPN HA series shows a q value in the range of 1.5–5.0, while the IPN HG series have q from 3.0–4.5. The influence of crosslinker concentration and type is reflected in the fact that an increase in EDC concentration leads to a decrease in the degree of swelling, and the introduction of NHS leads to even less swelling for both HA and HG series.

### 3.6. Hydrophilicity of Hydrogel Scaffolds

Scientific studies indicate that in vivo tissue compatibility as well as in vivo biofunctionality and medical device safety (including drug release systems) can be influenced by varying surface characteristics including hydrophilicity. Hydrophilicity is a very important property for biomedical applications of scaffolding biomaterials. The surface hydrophilicity of the obtained hydrogel scaffolds was evaluated by water surface contact angle tests. All HA and HG IPN hydrogel scaffolds series are fully hydrophilic (measurements were performed at 0 and 1 s), which means that water completely wetted surfaces and drops currently vanish after application on the surface of the hydrogel scaffolds. This behavior indicates that HA and HG hydrogel scaffolds series are favorable candidates as scaffolding polymeric biomaterials for biomedical uses.

### 3.7. Biocompatibility Assays of Hydrogel Scaffolds

#### 3.7.1. Effect of Hydrogel Scaffolds on Cytotoxicity and Cell Viability (MTT)

**Figure 4:**
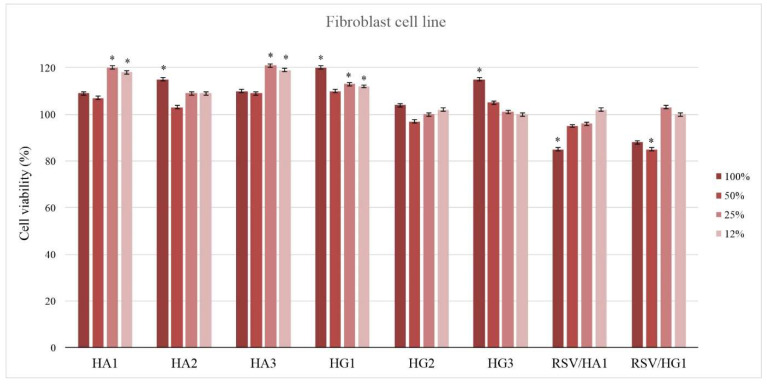
In vitro cytotoxicity (antiproliferative effect) profile of HA and HG hydrogel scaffolds series extracts against a human lung fibroblast (MRC5) cell line in the presence of 12%, 25%, 50%, and 100%, v/v of material extracts. (Percentage of alive cells was compared to the DMSO-treated control using a t-test; * *p* ≤ 0.01).

The biocompatibility of scaffolding biomaterials is a substantial feature to be considered for biomedical engineering applications. Cytotoxicity as a simple and fast preliminary project test is an important indicator for the toxicity assessment of medical devices. Three types of cytotoxicity tests are specified in the International Organization for Standardization 109993-5: extract, direct contact, and indirect contact. The in vitro cell viability of the HA and HG hydrogel scaffolds series and samples loaded with resveratrol as the active agent was tested on a healthy human fibroblast (MRC5) cell line treated with biomaterial extract (Figure 4). The prepared extracts of HA and HG hydrogel scaffolds series were obtained after extended shaking and immersion in RPMI medium at 37 °C for 3 days. HA and HG hydrogel scaffold series’ extracts were not cytotoxic in vitro under tested conditions, and lower doses of extracts resulted in higher proliferation in MRC5 cells compared to the untreated control, which was 100% (Figure 4). The stimulative effect on MRC5 cell proliferation was most noticeable when the lowest concentration of the material’s extract was applied (12%, v/v). Loading the bioactive agent resveratrol (RSV) in hydrogel scaffolds slightly decreases cell viability but certainly shows biologically accepted values. All tested hydrogel scaffold samples showed no cytotoxicity and favorable in vitro biocompatibility.

#### 3.7.2. In vivo Evaluation of Hydrogel Scaffolds Using *Caenorhabditis elegans* Survival Assay

Toxicity studies using mammalian models are robust, time-consuming, and expensive; therefore, as an intermediate between in vitro and mammalian testing, *Caenorhabditis elegans* emerges as an excellent alternative [89]. The nematode *C. elegans* has long been recognized as one of the premier model organisms for disease modeling, drug discovery, and toxicity assessments, due to multiple specialized tissues and a large number of conserved genes and signaling pathways that are shared with humans [90,91].

**Figure 5:**
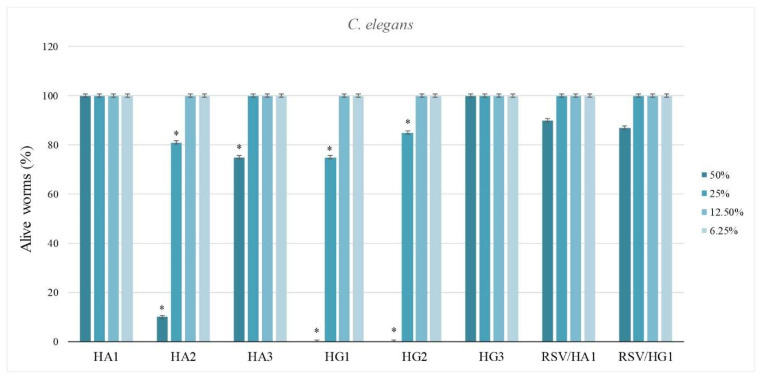
Nematodotoxicity of HA and HG hydrogel scaffold series for in vivo *Caenorhabditis elegans* survival assay in the presence of 12%, 25%, 50%, and 100% v/v of material extracts. (Percentage of alive worms was compared to the DMSO-treated control using a t-test, * *p* ≤ 0.01).

*C. elegans*, as a microworm, is used for in vivo assay to estimate biomedical performances of HA and HG hydrogel scaffolds series as well as samples’ loading with the bioactive agent resveratrol (RSV) [92,93,94]. Nematodotoxicity was observed at the highest concentrations for in vivo *C. elegans* survival assay. Samples HA1, HA3, and HG3 were safe even at the highest concentrations. Samples HA2, HG1, and HG2 were highly nematodotoxic at the highest concentration (50%) and moderately nematodotoxic at a concentration of 25% (Figure 5). It can be stated that the loading of RSV as a bioactive agent in hydrogel scaffolds increases the favorable in vivo response. Generally, there is an influence of composition, crosslinker concentration, and type of crosslinker on the in vivo behavior using interaction with *C. elegans*. Therefore, it can be said that obtained HA1, HA3, and HG3 hydrogel scaffolds show the best in vivo biocompatibility and significant potential for biomedical applications. The loading of resveratrol into hydrogel scaffolds improves in vivo biocompatible performances, which proves the bioactivity of this agent.

### 3.8. In Vitro Controlled Resveratrol Release Study

**Figure 6:**
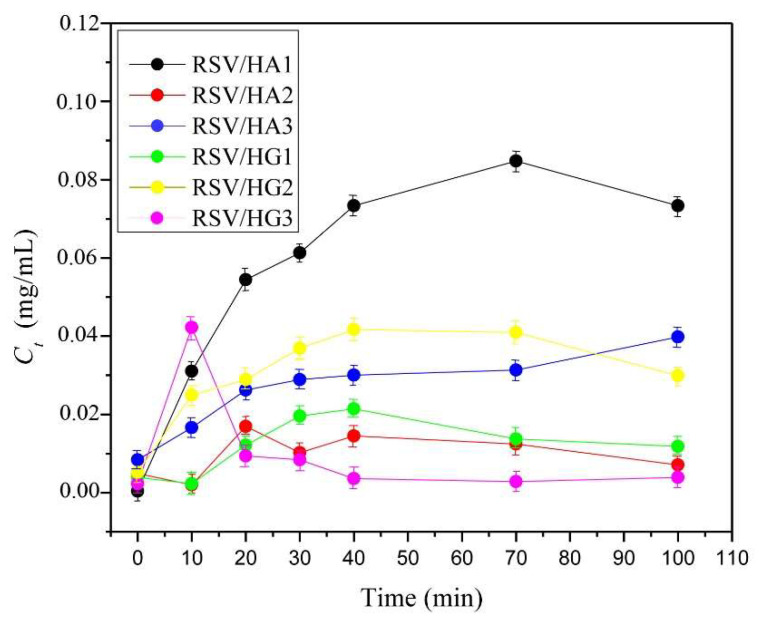
In vitro controlled resveratrol release profiles from hydrogel scaffolds.

In vitro and in vivo biocompatibility assays of the HA and HG series of hydrogel scaffolds confirmed that obtained hydrogel scaffolds series are safe for biomedical applications. An additional goal of this study is to evaluate their applicability as a platform for controlled bioactive agent resveratrol release. Resveratrol (RSV) is a natural, bioactive agent with great beneficial effects (cardioprotective, antioxidant, anti-inflammatory, anti-hyperlipidemic, and anti-obesity) [58,59,60,61,62,63,64,65,66,67,68,69,70,71,72,73]. To evaluate in vitro controlled resveratrol release potential from HA and HG hydrogel scaffolds series release study was performed in a phosphate buffer of pH 7.40, at 37 °C, for a shorter period of time. The results obtained (Figure 6) revealed a dependence of RSV release performances on scaffold composition (alginate or gelatin) and the concentration and type of crosslinker. The best potential for RSV release was shown by sample HA1, which is consistent with swelling, followed by the samples HG2 and HA3. The other samples show lower RSV release performances. The release process is a complex phenomenon that is influenced by the scaffold’s composition and the type of bioactive agent, the cross-links that have been created, and the release conditions. These release platforms are designed to release RSV in a short period of time, with the aim of application in dermal regeneration.

#### Parameters of Resveratrol Release from Hydrogel Scaffolds

Four kinetic models (Table 3) were applied to study the release kinetics of resveratrol from HA and HG hydrogel scaffolds series to obtain significant release parameters for these RSV/HA and RSV/HG scaffolding release platforms to give more extensive insight into the release process. The in vitro-obtained resveratrol release data were used to fit according to the kinetic models. Calculated parameters are presented in Table 4.

The RSV release process from synthesized IPN HA and HG series was evaluated by fitting the experimental data using four kinetic models (Higuchi, Ritger–Peppas, Peppas–Sahlin, and Peppas–Sahlin model when m = 0.5) to obtain characteristic release parameters for RSV/HA and RSV/HG scaffolding release platforms [95,96,97,98]. Experimental data were analyzed by nonlinear least-squares regression. The sum of the squared residuals (SSR) and Akaike Information Criterion (AIC) was determined for each model as indicators of the model’s suitability for the obtained data set. The model that shows the smallest value for the AIC best describes the resveratrol release process [95,96,97,98].

Based on the theoretical approach, Higuchi model is not suitable to analyze swellable drug release systems by itself, thus additional mathematical analysis must be performed [99]. Ritger–Peppas model is more convenient for that purpose because the only difference between these two models is the exponent n. Since Higuchi model assumes n = 0.5, resveratrol release from the hydrogels is best fitted when the n value obtained from Ritger–Peppas is close to 0.5. According to the obtained n values presented in Table 4, Higuchi model is not suitable for the interpretation of resveratrol release from the synthesized hydrogels. The n > 0.85 for RSV/HA1, RSV/HA2, and RSV/HG1 elucidate the case II transport, and for RSV/HA3, RSV/HG2, and RSV/HG3 n < 0.85 anomalous (non-Fickian) transport.

Peppas–Sahlin model was used for the calculation of the approximate contribution and coupled effect of Fickian diffusion and polymer relaxation mechanism to the release process. Kinetic constant k_1_ represents Fickian contribution, while k_2_ stands for polymer relaxation contribution (case II transport) to resveratrol release. According to the obtained values of kinetic constants (Table 4), Fickian contribution is dominant over the relaxation of polymer chains in the case of resveratrol release (k_1_ > k_2_). On the other hand, when the diffusion constant m is held at 0.50, higher values of k_2_ were obtained (Table 4) compared to k_1_ for RSV/HA1, RSV/HA2, and RSV/HG1, indicating case II transport and dominant contribution of polymer relaxation to resveratrol release mechanism, which is in accordance with the results obtained using Ritger–Peppas model.

Analyzing the Akaike information criterion (AIC) values, it turns out that Higuchi model fits for RSV/HG2 sample; Ritger–Peppas model fits for the RSV/HG3 sample; Peppas–Sahlin model fits the RSV/HA1, RSV/HA2, and RSV/HG1 samples; and Peppas–Sahlin when m = 0.5 fits for the RSV/HA3 sample (Table 4). It is obvious that the composition of the scaffold and the type and concentration of cross-linker influence the release process of RSV/HA and RSV/HG release platforms, which is reflected in the fact that different models give the best fit for the process of resveratrol release from the scaffolds.

## 4. Conclusions

Two series of new HEMA/alginate and HEMA/gelatin IPN scaffolds, designed as multifunctional, bioactive scaffolding for multiple biomedical purposes, including controlled release of the bioactive agent resveratrol, were successfully fabricated using free-radical polymerization/crosslinking reactions. The HA and HG samples were fabricated by varying concentrations and type of crosslinker (EDC and EDC/NHS), and their structural characteristics were confirmed by spectral analysis (FTIR spectra). All samples showed the presence of favorable interconnected porous morphology, while the shape of pores depended on the crosslinker concentration and type—ellipsoidal to spherical pore structure with EDC and honeycomb morphology with EDC/NHS. Generally, high values of Young’s modulus were obtained, depending on the scaffold’s composition and the used crosslinker and their concentration, which satisfy the mechanical strength needed for drug delivery systems. The swelling properties are in accordance with porosity values.

HA and HG hydrogel extracts were not cytotoxic in vitro and showed favorable biocompatibility tested using a healthy human fibroblast (MRC5) cell line. The stimulative effect on MRC5 cell proliferation was most noticeable with the lowest concentration of the material’s extract (12%, v/v). Loading the bioactive agent resveratrol into hydrogel scaffolds slightly decreased cell viability but showed biologically acceptable values.

In vivo evaluation of hydrogel scaffolds was performed using *Caenorhabditis elegans* survival assay to estimate the biomedical performances of all samples, as well as those of extracts of hydrogel samples loaded with the bioactive agent resveratrol (RSV). At the highest extract concentration (50%), nematodotoxicity was observed for samples HA2, HG1 and HG2, while moderate nematodotoxic was observed at a concentration of 25%. Samples HA1, HA3, and HG3 were safe even at the highest extract concentrations. The loading of RSV in hydrogel scaffolds increased the favorable in vivo response. Generally, there is an influence of composition, crosslinker concentration, and type of crosslinker on in vivo behavior using interaction with *Caenorhabditis elegans*.

Characteristic release parameters for RSV/HA and RSV/HG release mechanisms obtained using four kinetic models (Higuchi, Ritger–Peppas, Peppas–Sahlin, and Peppas–Sahlin model when m = 0.5) showed that sample RSV/HG3 exhibited the lowest values for SSR and AIC compared to other investigated hydrogels, implicating that this hydrogel could have the most appropriate physicochemical characteristics for the resveratrol release.

According to the experimental results for IPN samples regarding physicochemical characterization, mechanical properties, in vitro and in vivo assays, as well as for characteristic release parameters, HA1, HA3, and HG3 samples show the best in vivo biocompatibility and significant potential for biomedical applications. Therefore, they can be safely used as suitable medical devices for biomedical applications, especially controlled drug release.

## Figures and Tables

**Table 1:** Composition of IPN hydrogels and sample marks.

Sample	Component 1	Component 2	Crosslinker for HEMA	Crosslinker(s) for Gelatin/Alginate	Initiator/Activator
HA1	HEMA	Alginate	PEGDMA	EDC	PPS/TEMED
HA2	HEMA	Alginate	PEGDMA	EDC	PPS/TEMED
HA3	HEMA	Alginate	PEGDMA	EDC/NHS	PPS/TEMED
HG1	HEMA	Gelatin	PEGDMA	EDC	PPS/TEMED
HG2	HEMA	Gelatin	PEGDMA	EDC	PPS/TEMED
HG3	HEMA	Gelatin	PEGDMA	EDC/NHS	PPS/TEMED

**Table 2:** Values for porosity and Young’s modulus of the hydrogel scaffolds.

Sample	Porosity (%)	Young’s Modulus (MPa)
HA1	83.83 ± 3.57	7.50 ± 0.17
HA2	71.40 ± 3.25	3.50 ± 0.08
HA3	85.20 ± 3.77	5.00 ± 0.12
HG1	62.36 ± 3.03	1.40 ± 0.04
HG2	84.26 ± 3.63	2.50 ± 0.06
HG3	72.38 ± 3.27	3.50 ± 0.07

**Table 3:** Kinetic models used to fit experimental drug release data.

Kinetic Model	Equation	Parameters
Higuchi equation-describes the Fickian diffusion of the drug	F = k_H_t^1/2^	Where F is the fractional drug release, k_H_ is the kinetic constant and t is the release time.
Ritger-Peppas equation	F = k_1_t^n^	Where F is the fractional drug release, k_1_ is the kinetic constant, t is the release time, and n is the diffusional exponent
Peppas-Sahlin equation, which accounts for the coupled effects of Fickian diffusion and Case II transport	F = k_1_t^m^ + k_2_t^2m^	The first term of this equation represents the contribution of Fickian diffusion, and the second term refers to the macromolecular relaxation contribution to the overall release process.
Peppas-Sahlin equation where exponent *m* fixed to 0.5	F = k_1_t^1/2^ + k_2_t	Where F is the fractional drug release, k_1_ and k_2_ are kinetic constants, and t is the release time.

**Table 4:** Release parameters obtained for RSV/HA and RSV/HG scaffolding release platforms using Higuchi, Ritger–Peppas, Peppas–Sahlin, and Peppas–Sahlin when m = 0.5 models.

Higuchi	RSV/HA1	RSV/HA2	RSV/HA3	RSV/HG1	RSV/HG2	RSV/HG3
k_H_	0.0972	0.1006	0.0929	0.0812	0.1123	0.0708
SSR	0.0399	0.0235	0.0069	0.0264	0.0224	4.278 × 10^−4^
R^2^	0.6197	0.6954	0.8340	0.6017	0.6986	0.9355
AIC	−10.89	−13.00	−17.91	−12.53	−13.20	−29.03
**Riter-Peppas**	**RSV/HA1**	**RSV/HA2**	**RSV/HA3**	**RSV/HG1**	**RSV/HG2**	**RSV/HG3**
k_1_	0.0072	0.0153	0.0294	0.0058	0.0459	0.0958
n	1.2707	1.0601	0.8430	1.2773	0.7679	0.4089
SSR	0.0158	0.0109	0.0022	0.0128	0.0247	2.492 × 10^−4^
R^2^	0.8490	0.8591	0.9482	0.8067	0.6667	0.9624
AIC	−12.58	−14.08	−20.57	−13.42	−10.79	−29.19
**Peppas-Sahlin**	**RSV/HA1**	**RSV/HA2**	**RSV/HA3**	**RSV/HG1**	**RSV/HG2**	**RSV/HG3**
k_1_	−0.0672	−0.0182	0.0254	−0.0439	0.0469	0.0854
k_2_	0.0305	0.0221	0.0125	0.0232	0.0122	−0.0027
SSR	0.0124	0.0105	0.0024	0.0122	0.0261	2.693 × 10^−4^
R^2^	0.8814	0.8635	0.9431	0.8157	0.6489	0.9594
AIC	−13.55	−14.21	−20.20	−13.62	−10.59	−28.88
**Peppas-Sahlin m = 0.5**	**RSV/HA1**	**RSV/HA2**	**RSV/HA3**	**RSV/HG1**	**RSV/HG2**	**RSV/HG3**
k_1_	0.0646	0.0807	0.0075	0.0985	0.1315	0.1002
k_2_	−0.0311	−0.0389	−0.0011	−0.5081	−0.0635	1.911 × 10^−5^
m	1.990	1.990	1.661	−7.052 × 10^−4^	1.990	0.3898
SSR	0.0207	0.0112	0.0011	0.0335	0.0147	4.948 × 10^−4^
R^2^	0.8025	0.8545	0.9747	0.4954	0.8018	0.9254
AIC	−9.51	−11.96	−21.44	−7.59	−10.87	−24.45

## Data Availability

Not applicable.
